# Neutron diffraction and DFT studies of oxygen defect and transport in higher-order Ruddlesden–Popper phase materials†

**DOI:** 10.1039/d3ra01772a

**Published:** 2023-05-05

**Authors:** Mudasir A. Yatoo, Ieuan D. Seymour, Stephen J. Skinner

**Affiliations:** a Imperial College London, Department of Materials, Faculty of Engineering Exhibition Road London SW7 2AZ UK m.yatoo15@imperial.ac.uk; b EPSRC Centre for Doctoral Training in Advanced Characterisation of Materials Exhibition Road London SW7 2AZ UK

## Abstract

A series of higher-order Ruddlesden–Popper phase materials – La_3_PrNi_3_O_10−*δ*_, La_2_Pr_2_Ni_3_O_10−*δ*_ and LaPr_3_Ni_3_O_10−*δ*_ – were synthesised and investigated by neutron powder diffraction to understand the oxygen defect structure and propose possible pathways for oxygen transport in these materials. Further complimentary DFT calculations of the materials were performed to support the experimental analysis. All of the phases were hypostoichiometric and it was observed that the majority of the oxygen vacancies were confined to the perovskite layers, with a preference for equatorial oxygen sites. A particular preference for vacancies in O(1) and O(5) sites at high temperatures was observed from neutron diffraction measurements which were further complimented by DFT calculations wherein the vacancy formation energy was found to be lowest at the O(1) site. Also, a preference for a curved oxygen transport pathway around the NiO_6_ octahedra was observed which agrees with the published literature for Ruddlesden–Popper phase materials. Lattice parameters for all three compositions showed a linear increase with increasing temperature, but the increase was greatest in the *c* parameter while the *b* parameter showed only a slight increase when compared to the *a* parameter. The thermal expansion coefficient was calculated for all compositions and was found to be in the range 13.0–13.4 × 10^−6^ °C^−1^, which is compatible with the commonly used electrolyte materials for solid oxide fuel cells.

## Abstract 2

The plot of formation energy for oxygen vacancies at the O1–O5 sites in the *Bmab* primitive cell structures of La_4_Ni_3_O_10_, La_2_Pr_2_Ni_3_O_10_ and Pr_4_Ni_3_O_10_, showing oxygen vacancies prefer O1, O2 and O3 crystallographic sites in perovskite layers.

## Graphical abstract

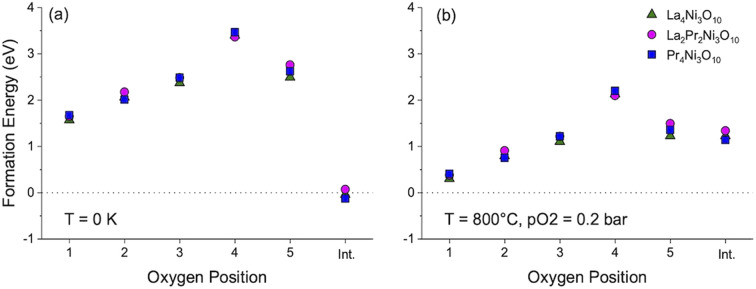

## 1 Introduction

Mixed ionic–electronic conductivity has been identified as one of the important properties of materials to be used as electrodes in solid oxide fuel cells (SOFCs) and electrolysers (SOECs), collectively termed Solid Oxide Cells (SOCs). As such, ionic conductivity plays an important role in determining the performance of oxygen ion conductors as electrodes in SOCs. Ionic conductivity is the movement of an ion from one site to another through defects in the crystal. In oxygen ion conductors such as the Ruddlesden–Popper (RP) phase materials where the composition is A_*n*+1_B_*n*_O_3*n*+1_, A = Ln and B = transition metals (*n* = 1–3), it is the oxygen defects (vacancies and/or interstitials) which determine the ionic conductivity because ionic conductivity of a material is directly proportional to the concentration of lattice defects. In this context, the investigation of the oxygen defects in these materials assumes importance.

It is here that neutron powder diffraction (NPD) plays a crucial role because the neutron scattering length of an oxygen atom is sizeable, 5.805 ± 0.004 fm (ref. 1) and thus easily detectable by neutron powder diffraction. Neutron diffraction is a reliable method to determine the crystallographic positions of the oxide ions, their atomic displacement parameters and their occupancies, while X-rays have a low sensitivity to oxygen, particularly in a matrix of heavy atoms. This could be later used to understand the oxygen conduction mechanisms. All of these parameters are of particular interest in materials with high oxygen mobility such as the Ruddlesden–Popper phases. NPD can also be used to understand the lattice parameter evolution of electrode materials to obtain information such as the thermal expansion coefficient (TEC) of materials, a critical mechanical aspect for the use of these materials in high-temperature devices such as SOCs.

Ruddlesden–Popper phases were first synthesised by Ruddlesden and Popper in 1958.^2^ The structure consists of *n*ABO_3_ perovskite layers which are sandwiched between two AO rock-salt layers.^3^ The lower-order (*n* = 1) phases such as La_2_NiO_4+*δ*_ (LNO) and Pr_2_NiO_4+*δ*_ (PNO) have been studied extensively.^4–7^ The main motivation for intense research focus on the *n* = 1 Ruddlesden–Popper phase materials stemmed from their superior capability of storing a substantial amount of interstitial oxygen in their structure which bestowed the materials with significant oxide ion conductivity, thereby rendering the materials mixed ionic–electronic conductors (MIEC) at intermediate temperatures.^8,9^

There has been an interesting and long debate on the nature of ionic conductivity in these materials. Therefore, detailed studies have been carried out on the LNO material and its transport properties are well understood. There is broad agreement regarding the dominant participation of interstitial oxygen atoms in ionic conductivity.^10^ The neutron diffraction studies by Demourgues *et al.* predicted that interstitial oxygen atoms are accommodated by the LaO rock salt layer.^11^ This was further confirmed by Jorgensen^12^ and co-workers and they determined the location of the oxygen interstitial site to be at 
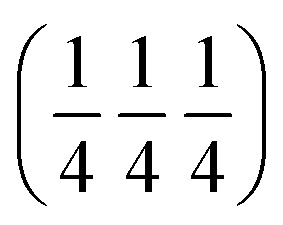
 in the *Fmmm* structure. These studies had further support from Paulus *et al.* who studied single crystals of LNO and confirmed the location of excess oxygen in the rock salt LaO layer, thereby lending further support to the initial studies.^13^ In light of these conclusions, Skinner observed the lengthening of apical Ni–O bonds on heating LNO, which is a direct consequence of the loss of excess oxygen from interstitial sites located in the La–O plane.^14^

Higher-order Ruddlesden–Popper phases, however, tend to be understoichiometric and, therefore, oxygen vacancies are dominant defects. In comparison to lower-order Ruddlesden–Popper phases, there are very few neutron diffraction studies of lanthanide-based higher-order Ruddlesden–Popper phases such as La_0.3_Sr_2.7_CoFeO_7−*δ*_ (*n* = 2), and LaSr_3_Co_1.5_Fe_1.5_O_10−*δ*_ (*n* = 3) available in the literature.^15–17^ This report will, therefore, detail our recent neutron diffraction studies and complimentary DFT calculations of three *n* = 3 Ruddlesden–Popper compositions – La_3_PrNi_3_O_10−*δ*_ (L3P1N3), La_2_Pr_2_Ni_3_O_10−*δ*_ (L2P2N3) and LaPr_3_Ni_3_O_10−*δ*_ (L1P3N3) – to provide a comprehensive study of oxygen defect structure and transport properties in these materials. The higher-order phases tend to be oxygen understoichiometric in nature and therefore oxygen vacancies are predominant defects in these materials. The three phases under discussion too were previously reported by our group to be oxygen deficient in nature.^6,18,19^

## 2 Methods

### 2.1 Syntheses and characterisation

All three compositions – L3P1N3, L2P2N3 and L1P3N3 – were synthesised using the sol–gel route.^20^ Stoichiometric quantities, depending on the material composition, of La(NO_3_)_3_·6H_2_O (Sigma Aldrich, 99.0%), Pr(NO_3_)_3_·6H_2_O (Sigma Aldrich, 99.99%) and Ni(NO_3_)_2_·6H_2_O (Sigma Aldrich, 99.0%) were dissolved in an aqueous solution of 10% (by weight) of citric acid (Sigma Aldrich, 99.99%). The solution was heated at 250 °C, under constant stirring for three hours, until a gel was obtained. The gel, after being decomposed in the air for 12 hours at 600 °C, was ground and the resultant powder was annealed for 24 hours in air at 950 °C (L1P3N3) and 1000 °C (L2P2N3 and L3P1N3) prior to phase determination by X-ray powder diffraction. The XRD patterns of the material were collected using a PANalytical X'Pert Pro MPD (Cu K_α_ source) and Rietveld refinement of the XRD data, performed with the GSAS/EXPGUI software package^21,22^ was used to confirm the phase identification. We recently reported the initial XRD studies detailing all three compositions.^23^

### 2.2 Neutron powder diffraction of L2P2N3 at POLARIS

*In situ* neutron powder diffraction data were collected on the high-flux medium resolution POLARIS diffractometer at ISIS, the UK spallation source at the Rutherford Appleton Laboratory, Oxfordshire, UK.^24^ A powder sample of L2P2N3 was loaded in a 4 cm high vanadium can and initial measurements were recorded at ambient temperature. Two thermocouples were attached to opposite sides of the sample container to control and monitor the furnace temperature. After calibration, diffraction data were collected at room temperature (25 °C) and 600 °C for ∼8 hours (150 μAmps), and every 50 degrees from 100 °C to 550 °C for ∼2 hours (25 μAmps) after equilibrating the temperature at each measurement. The sample was kept under a vacuum at all times. All the data were collected in the *d*-spacing range of 0.40–4.50 Å and the data were corrected for absorption before analysing by Rietveld refinement.

### 2.3 Neutron powder diffraction of L1P3N3 and L3P1N3 at POWGEN

*In situ* neutron powder diffraction measurements were also collected for the other two compositions, L1P3N3 and L3P1N3 at the POWGEN beamline (0.0008 < Δ*d*/*d* < 0.025), SNS, Oak Ridge National Laboratory, USA.^25,26^ Powder samples were placed in a quartz sample holder with a 0.5 cm radius and 4 cm length. A fused silica quartz tube furnace, with vanadium foil elements operating under vacuum conditions, was used to heat the sample. A quartz line was used as a vertical hanger for holding the quartz sample holder, which was then lowered into the quartz tube until the sample was in the path of the beam and the furnace hot zone.^27^ The furnace temperature was calibrated using a ZnO reference sample.^28^ Powder samples were equilibrated under a continuous flow of simulated air (20% O_2_) for at least one hour before measurement at each temperature. Diffraction patterns were also collected every 100 °C from room temperature to 800 °C in the *d*-spacing range of 0.45–5.35 Å. Several patterns with shortened collection times were collected and examined prior to the 2 hour-long measurements to ensure that the sample had reached equilibrium, monitored by means of observing any shifts in lattice parameters.

It should be noted that the neutron powder diffraction data recorded on the POLARIS instrument were in a vacuum, whilst the data collected on the POWGEN diffractometer were in a synthetic air atmosphere. Evidently, the vacuum atmosphere, particularly at elevated temperatures, could induce reduction (oxygen loss), and hence direct comparisons of the oxygen contents of the three materials are not possible.

### 2.4 DFT calculations

Spin polarised density functional theory (DFT) calculations were performed in this work with the Vienna *Ab initio* Simulation Package (VASP), using the Perdew–Burke–Ernzerhof (PBE) exchange–correlation functional.^29,30^ Projector-augmented wave (PAW) pseudopotentials were used for all species.^31^ Two sets of pseudopotentials were used throughout this work. For the convex hull analysis of La_*x*_Pr_4−*x*_Ni_3_O_10_ structures, a softer set of PAW pseudopotentials was used, labelled La, Ni, and O_s in the VASP 5 distribution, using a plane wave cut off of 400 eV. Previous studies have shown that Pr adopts the Pr^3+^ oxidation state in Pr_4_Ni_3_O_10_.^32^ The Pr_3 pseudopotential in VASP was used in which the 4f electrons are moved to the core to alleviate the known issues with overbinding associated with f electrons. For the calculation of structural properties, defect formation energies and activation energies, p valence electrons were included for Ni (Ni_pv) and the normal oxygen pseudopotential (O) was used. For this later pseudopotential set, a plane wave cut off of 520 eV was used. An electronic convergence of 1 × 10^−6^ eV was used for all calculations. A Hubbard *U* parameter was added to Ni using the rotationally invariant approach of Dudarev *et al.*, to improve the description of electron correlation.^33^ A *U* value of 6.2 eV was used for Ni, taken from the Materials Project database.^34^ This value is calculated based on the approach of Wang *et al.*^35^ using the experimental formation energies of the reaction Li_2_O + 2NiO + 1/2O_2_ → LiNiO_2_,^31^ which captures the Ni^2+^/Ni^3+^ redox couple. Gamma centred *k*-point meshes were used for all calculations to sample the Brillouin zone with a *k*-point grid density of at least 20 Å. A collinear ferromagnetic spin alignment was used for all calculations in this study.

A *Bmab* unit cell structure was used for all calculations of La_*x*_Pr_4−*x*_Ni_3_O_10_ structures. Initial test calculations were performed for La_4_Ni_3_O_10_ in the higher symmetry *I*4/*mmm* structure, however, the structure relaxed to the *Bmab* structure upon full optimisation of the atomic positions and unit cell parameters. All distinct La/Pr orderings within the primitive *Bmab* unit cell structure (2 formula units) of La_*x*_Pr_4−*x*_Ni_3_O_10_ for *x* = 0, 1, 2, 3 and 4 were enumerated using the CASM package, to predict the formation energy of phases on the convex energy hull.^36^ The atomic positions and unit cell parameters of all structures were fully optimised without symmetry constraints until the force on any atom fell below 0.05 eV Å^−1^.

The Pr occupancy (*X*_M(*j*)_) on the two lanthanide sites within the structure, M(1) and M(2), was approximated by taking a Boltzmann average of all configurations at each composition, *x*:
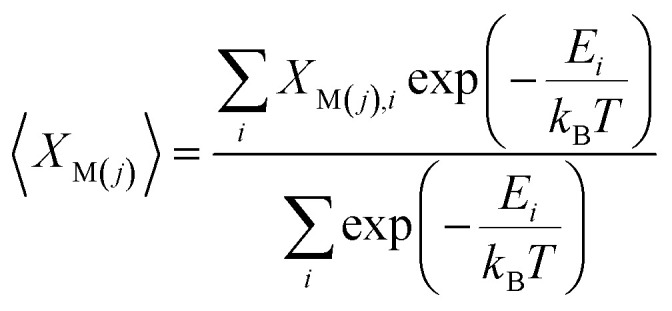
where *E*_*i*_ is the energy of structure *i*, *T* is the temperature and *k*_B_ is Boltzmann's constant.

For defect formation energy calculations, a single oxygen vacancy defect was introduced into 5 distinct oxygen positions (O1–O5) of the primitive *Bmab* unit cell structures of La_8_Ni_6_O_20±1_, Pr_8_Ni_6_O_20±1_ and the lowest energy structure of La_4_Pr_4_Ni_6_O_20±1_ found from the convex hull analysis. An oxygen interstitial defect was also introduced into each structure within the (La,Pr)O rocksalt layer. The atomic positions and lattice parameters for each structure were allowed to fully relax to a force tolerance of 0.01 eV Å^−1^. The chemical potential of oxygen (*μ*_O_2__) in the defect calculations was referenced to O_2_ gas *via* the calculation of the energy of and isolated triplet O_2_ molecule (*E*_O_2__) in a box (12 × 12 × 12 Å). To correct for the known over binding of O_2_ molecules with the PBE functional, a correction of +1.374 eV was added to the energy of O_2_, based on the procedure previous described by Wang *et al.*^35^ The impact of temperature on the oxygen chemical potential, *μ*_O_2__(*T*,*p*_O_2__), was approximated as:^37^
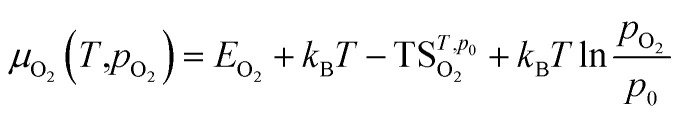
where 
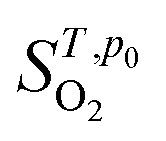
 is the entropy of the O_2_ molecule taken from the JANAF thermochemical tables,^38^*p*_O_2__ is the partial pressure of O_2_ gas and *p*_0_ is the partial pressure of oxygen in the standard state. Structure figures were produced using the VESTA software package.^39^

The activation barriers for vacancy diffusion between adjacent O1 sites within the La_4_Ni_3_O_10_, Pr_4_Ni_3_O_10_ and L2P2N3 structure were calculated using the climbing image nudged elastic band approach (CI-NEB).^40^ To minimise the interactions between vacancies in adjacent cells, 2 × 2 × 1 supercells of the initial primitive *Bmab* unit cells were created. A single vacancy was introduced into the supercell (M_32_Ni_24_O_79_) into neighbouring O1 sites, and the atomic positions were optimised under fixed cell conditions to a force tolerance of 0.01 eV Å^−1^. CI-NEB^40^ were performed with 5 intermediate images under fixed cell condition using same force tolerance.

## 3 Results and discussion

### 3.1 Symmetry and evolution of the lattice constants

The evolution of unit cell constants, and thereby the unit cell volume is an important consideration for these materials as they are considered for potential use in high-temperature SOC devices. Therefore, Rietveld refinements, to be discussed in detail in the next section, were performed using the GSAS/EXPGUI software package^15,16^ and it was found that L1P3N3 and L3P1N3 compositions studied by POWGEN crystallised in the low-symmetry orthorhombic space group *Bmab* at all temperatures except the L3P1N3 composition which adopts the high-symmetry tetragonal space group *I*4/*mmm* at 800 °C. L2P2N3 studied with the POLARIS diffractometer, however, crystallised with monoclinic symmetry. Table 1 summarises the room-temperature lattice constants obtained from Rietveld refinement of neutron diffraction data for the L3P1N3, L2P2N3 and L1P3N3 compositions and compares them with the two end members as reported by Zhang *et al.*^41^ The cell constant data obtained from the NPD measurements are in excellent agreement with our previous XRD studies.^23^

**Table 1:** Unit-cell parameter comparison of the three prepared compositions: L1P3N3, L2P2N3 and L3P1N3. The two endmembers, L4N3 and P4N3, are also included to show the systematic decrease of lattice parameters with increasing Pr content, which is consistent with the variation of the ionic radii leading to an overall decrease in the cell volume in going from L4N3 to P4N3

Composition	*a* (Å)	*b* (Å)	*c* (Å)	Space group	Reference	Instrument
La_4_Ni_3_O_10−*δ*_	5.415(1)	5.465(1)	27.959(9)	*Fmmm*	Zhang *et al.*^36^	XRD
L3P1N3	5.41027(7)	**5.46975(7)**	27.8955(4)	*Bmab*	This work	POWGEN
L2P2N3	5.38773(8)	5.46047(8)	27.7533(5)	*P*2_1_/*a*	This work	POLARIS
L1P3N3	5.38686(1)	**5.46975(8)**	27.7463(7)	*Bmab*	This work	POWGEN
Pr_4_Ni_3_O_10−*δ*_	5.370(1)	5.462(1)	27.528(3)	*Fmmm*	Zhang *et al.*^36^	XRD

**Fig. 1:**
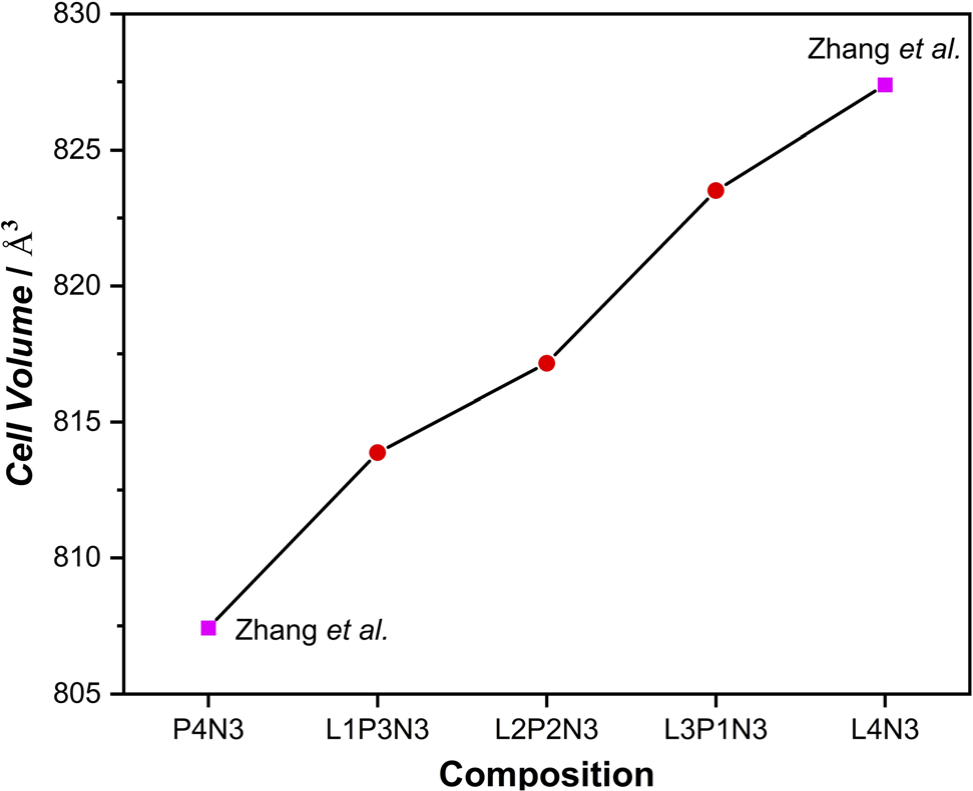
Cell volume increase with the increase in La content of the composition, reflecting the trend observed in the unit cell constant evolution. (The cell volumes plotted run up to 2 significant digits and the estimated std. dev. lie within the data markers.)

As is evident from the data in Table 1, a decrease in unit cell constants with increasing Pr content was observed. This is consistent with the decrease in the ionic radius of Pr leading to an overall decrease in the cell volume. This agrees with our X-ray diffraction studies of these materials^6,23^ and the other published literature^41,42^ for the end member compositions. The significant decrease observed in the *c* parameter with increasing Pr content is explained by the fact that there is a cumulative effect of reduced lanthanide radii along the crystallographic *c* direction where the layers are stacked. The overall decrease in cell parameters in going from L4N3 to P4N3 is reflected in the unit cell volume as a function of composition, Fig. 1. There, however, appears to be a very slight increase in *b* parameter in L3P1N3 and L1P3N3 compositions (shown in bold) when compared with the respective preceding compositions which go against the expected trend. This could be because the L3P1N3 and L1P3N3 measurements (POWGEN) presented above were obtained at slightly higher temperatures, 70 °C while L2P2N3 was measured at 25 °C (POLARIS).

In recent high-resolution X-ray and neutron diffraction studies,^43–46^ higher-order Ruddlesden–Popper phases have been shown to adopt monoclinic symmetry. With this in mind, we investigated the POLARIS high-resolution detector, bank 5 (Δ*d*/*d* = 3 × 10^−3^) L2P2N3 composition data set and found that the material adopts the monoclinic *P*2_1_/*a* space group symmetry at room temperature, Fig. S1.† The lattice parameters obtained were *a* = 5.38773(8) Å, *b* = 5.46047(8) Å, *c* = 27.7533(5) Å with the cell angles obtained being: *α* = *γ* = 90.0 and *β* = 90.235(2). It is evident that the monoclinic distortion of the cell is slight and is only observed when the highest resolution data are obtained. It is therefore likely that the orthorhombic assignment of the lower temperature data reported here and in the literature is a feature of the instrument resolution and that the true symmetry is monoclinic. For consistency, however, all further data are related to the orthorhombic cell identified with the lower resolution instruments.

### 3.2 Thermal expansion coefficients

**Fig. 2:**
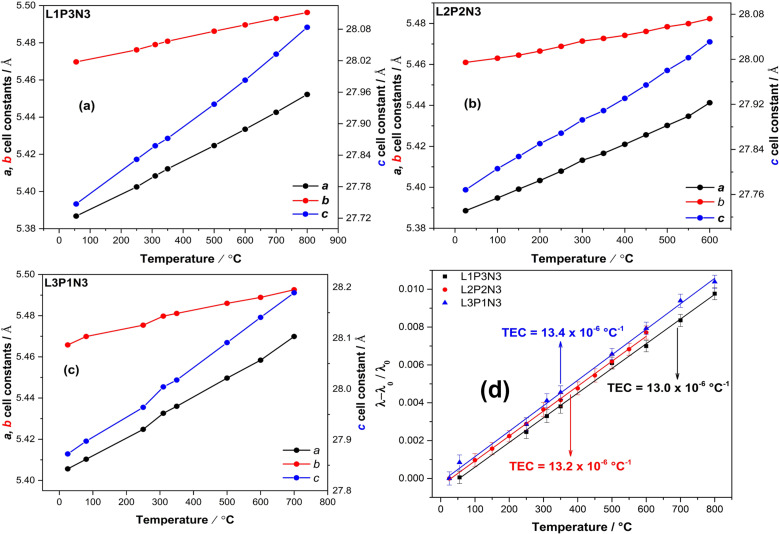
Increase of the unit cell parameters with the increase in temperature for (a) L1P3N3 composition (b) L2P2N3 composition and (c) L3P1N3 composition. Unit cell constant *c* shows a significant change, reflective of the cumulative effect of stacking of layers across crystallographic *c* direction. (d) (*λ*–*λ*_0_/*λ*_0_) as a function of temperature for all the three compositions showing the compatible TEC's. (Note: standard deviations for the unit cell parameters lie within the data markers.)

Fig. 2a–c illustrate the increase of the *a*, *b* and *c* lattice parameters as a function of increasing temperature and Fig. 2d shows the calculated thermal expansion coefficients (TEC) for all three compositions. As expected, the unit cell parameters show a linear increase throughout the temperature range. On close examination of the data, it is evident that the *c* parameter showed a significant increase when compared to both the *a* and *b* parameters, thereby reflecting the cumulative effect of stacking of layers across the crystallographic *c* direction. The *c* parameter was more responsive to the changes in temperature than the *a* parameter because the expansion along the *c* axis is less restricted by the expansion of the rock-salt layers.^16,47^ Also evident from Fig. 2 is the relatively higher increase in *a* lattice parameter compared with the *b* lattice parameter, which points towards an increase in orthorhombic strain with increasing temperature as argued by us in our earlier reports.^23,48^

The slope of (*λ*–*λ*_0_/*λ*_0_) *vs. T* (Fig. 2d), where *λ* and *λ*_0_ are the cubic roots of the volume at a particular temperature and room temperature respectively were used to calculate thermal expansion coefficients. The calculated values of the TEC were found to be 13.0 × 10^−6^ °C^−1^ for L1P3N3; 13.2 × 10^−6^ °C^−1^ for L2P2N3 and 13.4 × 10^−6^ °C^−1^ for L3P1N3, of the same order of magnitude as those of the other components of SOFCs, and therefore compatible with commonly used electrolytes such as Y_2_O_3_-stabilised ZrO_2_ (YSZ), Ce_0.8_Gd_0.2_O_2−*δ*_ (CGO) and La_0.8_Sr_0.2_Ga_0.8_Mg_0.2_O_3−*δ*_ (LSGM).^49,50^ These results, however, vary slightly from the value, 10 × 10^−6^ °C^−1^, calculated from XRD measurements and reported by Vaibhav *et al.* for P4N3 composition.^51^ It could possibly of different La : Pr ratio and the fact that they measured the TEC from room temperature to 1000 °C while the results reported here were measured over the temperature range of RT to 800 °C for L1P3N3 and L3P1N3 compositions and RT to 600 °C for L2P2N3 composition.

### 3.3 La/Pr ordering: neutron diffraction and DFT calculations

Complimentary DFT calculations were performed to support the experimental analysis. DFT calculations on unit cell structures of La_*x*_Pr_4−*x*_Ni_3_O_10_ (*x* = 0–4) were used to investigate the energetic preference for La/Pr ordering.

**Fig. 3:**
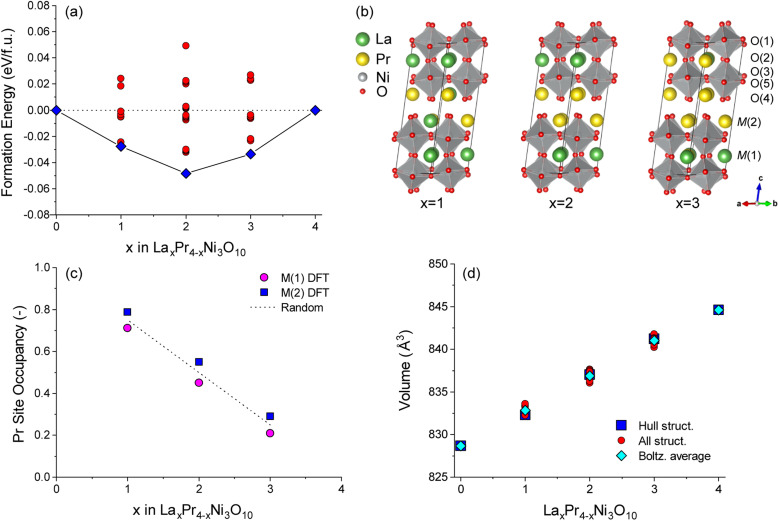
(a) Convex energy hull for La_*x*_Pr_4−*x*_Ni_3_O_10_ structures calculated with DFT. Both the lowest energy structures on the hull (blue squares) and configurations above the hull (red circles) are shown. (b) Lowest energy hull structures for *x* = 1, 2 and 3. (c) Boltzmann averaged Pr occupancy on M(1) (perovskite) and M(2) (rocksalt) sites as a function of composition, *x*. Occupancies are calculated at 800 °C from all convex hull structures at each composition. The random solution limit is shown as a dashed line. (d) Comparison of unit cell volume with composition, *x*, for all structures in the convex hull (red circles). The unit cell volumes of the lowest energy structures as shown as blue squares and the Boltzmann averaged unit cell volume for each composition at 800 °C is shown as cyan diamonds.

The energies of all symmetrically distinct La/Pr orderings on the M(1) (perovskite) and M(2) (rocksalt) sites were calculated with DFT+*U* using the *Bmab* unit cell of La_*x*_Pr_4−*x*_Ni_3_O_10_. The resulting convex energy hull is shown in Fig. 3a. Compositions of LaPr_3_Ni_3_O_10_ (*x* = 1), La_2_Pr_2_Ni_3_O_10_ (*x* = 2) and La_3_PrNi_3_O_10_ (*x* = 3) are observed as stable phases on the hull. The lowest energy ordering for each structure are shown in Fig. 3b. In all of the low energy structures on the hull, Pr preferentially occupies the M(2) site in the rocksalt layer.

To gauge the preference for La/Pr ordering at the experimental measurement temperature of 800 °C, a Boltzmann average of the Pr site occupancy was calculated from the cell energies of all structures at each composition, as shown in Fig. 3c. For all *x* compositions, at 800 °C there is only a weak preference for Pr ordering on the M(2) site, as the thermal energy (*k*_B_*T* = 0.107 eV) is larger than the energy difference between most of the configurations. The occupancy of the M(1) and M(2) sites is therefore close to the random solution limit of 0.5 : 0.5.

The Rietveld refinements of the neutron diffraction measurements revealed that the fractional occupancy for the A-site cations, La and Pr, were slightly different, than the nominal compositions; La_3.26_Pr_0.74_Ni_3_O_10−*δ*_, La_1.9_Pr_2.1_Ni_3_O_10−*δ*_ and La_1.04_Pr_2.96_Ni_3_O_10−*δ*_. Furthermore, the examination of the data for 50 : 50 composition (L2P2N3) in Tables 2 and 3 shows that Pr preferentially orders into the 9-coordinated M(2) site in the rocksalt layer as compared to the 12-coordinated M(1) in the perovskite layer. This further agrees with our DFT calculations discussed above (Fig. 3c). However, in contrast to the DFT calculations showing only a weak preference for Pr ordering on the M(2) site for all the compositions, the neutron diffraction measurements of L1P3N3 and L3P1N3 compositions at elevated temperatures showed that Pr has a weak preference for M(1) site.

**Table 2:** Rietveld refinement parameters obtained for the L2P2N3 composition recorded at 25 °C with an orthorhombic model (*Bmab*) (*χ*^2^ = 2.2; *R*_p_ = 4.2% and *R*_wp_ = 2.5%)

Atom	Atomic displacement parameters (Å^2^) × 100					Wyckoff position	Fractional occupancy
	*U*_11_/*U*_iso_	*U*_22_	*U*_33_	*U*_12_	*U*_23_		
La(1)	0.6(9)					8f	0.57(3)
Pr(1)	0.6(9)					8f	0.43(3)
La(2)	0.4(1)					8f	0.38(4)
Pr(2)	0.4(1)					8f	0.62(4)
Ni(1)	0.4(1)					4a	1.0
Ni(2)	0.4(1)					8f	1.0
O(1)	4.0(5)	3.8(4)	0.8(4)	4.7(3)		8e	0.98(3)
O(2)	0.4(2)	0.3(2)	2.5(3)		0.5(4)	8f	1.0
O(3)	0.1(3)	0.8(3)	1.3(3)	0.4(2)		8e	1.0
O(4)	1.6(3)	3.5(5)	0.4(2)		1.8(6)	8f	1.0
O(5)	1.5(3)	1.0(3)	0.2(2)	0.2(2)		8e	1.0

**Table 3:** Rietveld refinement parameters obtained for the L2P2N3 composition recorded at 600 °C with an orthorhombic model (*Bmab*) (*χ*^2^ = 3.6; *R*_p_ = 3.8% and *R*_wp_ = 2.1%)

Atom	Atomic displacement parameters (Å^2^) × 100					Wyckoff position	Fractional occupancy
	*U*_11_/*U*_iso_	*U*_22_	*U*_33_	*U*_12_	*U*_23_		
La(1)	2.2(1)					8f	0.57(3)
Pr(1)	2.2(1)					8f	0.43(3)
La(2)	0.9(1)					8f	0.38(4)
Pr(2)	0.9(1)					8f	0.62(4)
Ni(1)	1.4(9)					4a	1.0
Ni(2)	1.2(6)					8f	1.0
O(1)	5.1(6)	4.1(6)	7.5(8)	5.4(4)		8e	0.92(2)
O(2)	2.9(4)	2.3(4)	3.6(4)		0.5(4)	8f	0.95(2)
O(3)	0.5(3)	1.7(4)	1.3(3)	0.3(2)		8e	1.0
O(4)	4.0(4)	4.8(7)	2.0(3)		1.8(6)	8f	0.99(1)
O(5)	3.0(6)	2.9(5)	0.6(4)	0.2(3)		8e	0.91(3)

The variation in the unit cell volume was calculated for each structure as shown in Fig. 3d. An approximately linear increase in the unit cell volume is observed from compositions of *x* = 0 to *x* = 4 in La_*x*_Pr_4−*x*_Ni_3_O_10_, which is consistent with the experiment (Fig. 1). A slightly larger unit cell volume is predicted computationally compared to the experiment, as is commonly observed with DFT(+*U*) calculations using the PBE functional. For all configurations studied, it can be seen from Fig. 3d that the change in the chemical composition (*x*) has a bigger influence on the unit cell volume than the La/Pr ordering at fixed composition.

### 3.4 Phase transition

**Fig. 4:**
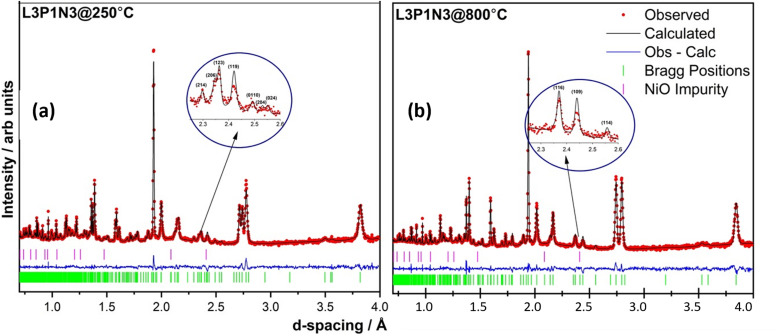
Rietveld refinement of the two data sets obtained at (a) 250 °C (*χ*^2^ = 4.297; *R*_p_ = 5.2% and *R*_wp_ = 4.3%) and (b) 800 °C (*χ*^2^ = 5.03; *R*_p_ = 5.8% and *R*_wp_ = 4.9%). A ∼2% impurity of NiO was detected and was refined in cubic *Fm*3̄*m* space group. The transition to a tetragonal high symmetry structure is easily discernible after examining the 2.3–2.6 Å *d*-spacing range as shown in the insets.

The neutron diffraction pattern obtained at 250 °C in flowing air for the L3P1N3 composition fitted best to an orthorhombic structure adopting the *Bmab* space group. The lattice parameters were determined to be *a* = 5.42478(2) Å, *b* = 5.47534(2) Å and *c* = 27.9632(1) Å, and are in close agreement with the previously reported results for the end member materials.^6,18,19,41,52^ A phase transition was observed on further heating and the diffraction pattern collected at 800 °C was refined with the tetragonal space group *I*4/*mmm*. The lattice parameters obtained after refinement again agree well with the literature for the end member composition and were *a* = *b* = 3.87876(1) Å, and *c* = 28.2287(2) Å.^16^ An impurity of ∼2 wt% NiO was also detected in the neutron diffraction data, which was not observed by X-ray diffraction measurements. The refinements of the data obtained at both temperatures, 250 and 800 °C, along with *χ*^2^ and *R* factors are illustrated in Fig. 4. The transition to the tetragonal *I*4/*mmm* high symmetry structure at 800 °C in flowing air is clearly visible after scrutiny of the 2.3 to 2.6 Å *d*-spacing range (insets, Fig. 4a and b). A close inspection of the insets confirms the assignment of the two structural models (*Bmab* and *I*4/*mmm*) at low and high temperatures respectively. The low-symmetry *Bmab* structure can be seen to show seven distinct reflections in the *d*-spacing range of 2.3–2.6 Å while the high-symmetry *I*4/*mmm* structure only shows three reflections in the same *d*-spacing range, thereby confirming the completion of the phase transition. The values of *x*, *y*, *z* and *U*_iso_ parameters determined by Rietveld refinement are provided in Tables S1 (250 °C) and S2 (800 °C) of the ESI.†

**Fig. 5:**
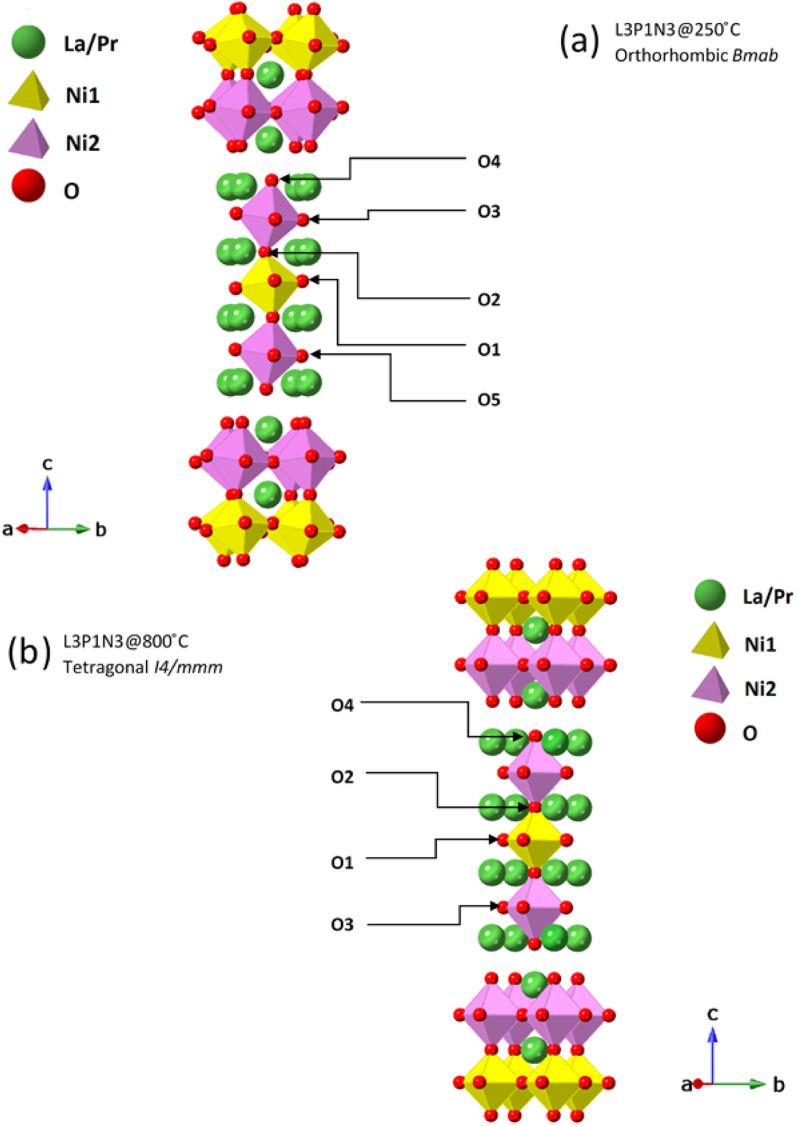
Ruddlesden–Popper unit cells for L3P1N3 structure. These structures were derived from Rietveld refinement of the neutron diffraction data collected at (a) 250 °C and (b) 800 °C. The tilt in the NiO_6_ octahedra in the 250 °C data set is clearly visible. Oxygen positions are further marked to guide the reader to visualise the oxygen transport pathways discussed.

It is instructive to examine the refined structures in detail; the structural changes on the transition from the orthorhombic to tetragonal structure primarily reflect changes in the NiO_6_ octahedra as demonstrated in Fig. 5. The tilt in the NiO_6_ octahedra for the sample measured at 250 °C is clearly visible when compared to the tetragonal structure obtained from the data set obtained at 800 °C. Similarly, the other two compositions, L2P2N3 (Fig. S2†) and L3P1N3 (Fig. S3†) were best refined in orthorhombic *Bmab* symmetry at both low and high temperature, and no transition to higher symmetry structures on heating was observed in either of the two compositions. However, as mentioned above L2P2N3 adopts monoclinic symmetry at RT, so it appears that there is a transition to higher symmetry orthorhombic structure at 250 °C, at least for the L2P2N3 composition.

The Ni(1)–O(2) and Ni(2)–O(4) bond lengths are instructive; the bond lengths determined from the data obtained at 250 °C for the L3P1N3 composition were 1.938(9) Å and 2.121(9) Å respectively. The main reason for this large difference in the bond distances is the fact that O(4) is the apical O atom shared between both the perovskite layer and the rock salt layer structural units in these RP phases and is therefore exposed to competing crystal-chemical driving forces. In fact, the elongation of the Ni–O bond involving the apical O atom which projects into the rock-salt type layer is found throughout the Ruddlesden–Popper series. This further points to the preference of Ni^3+^ occupying the Ni(1) site and Ni^2+^ occupying the Ni(2) site and this kind of charge ordering have indeed been reported earlier for Ruddlesden–Popper phase materials.^53^ Similar behaviour was observed in this work for the L2P2N3 and L1P3N3 compositions.

### 3.5 Oxygen defects and transport: neutron diffraction and DFT calculations

Atomic displacement parameters and fractional occupancies obtained after refinement of neutron diffraction data of the representative L2P2N3 composition, along with *χ*^2^ and *R* factors are illustrated in Tables 2 and 3 below.

**Fig. 6:**
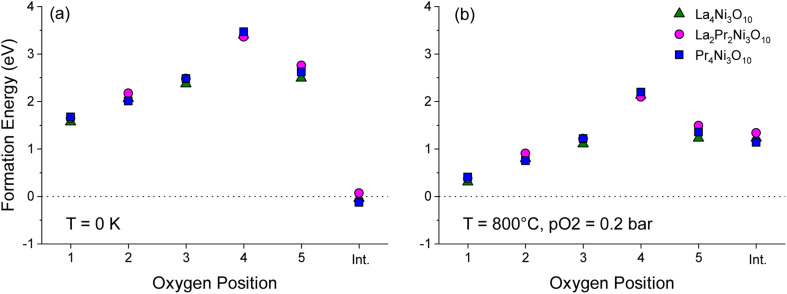
Plot of formation energy for oxygen vacancies at the O1–O5 sites in the *Bmab* primitive cell structures of La_4_Ni_3_O_10_, La_2_Pr_2_Ni_3_O_10_ and Pr_4_Ni_3_O_10_, with 2 formula units per cell (M_8_Ni_6_O_20±1_) at (a) *T* = 0 K and *p*_O_2__ = 0 bar and (b) *T* = 800 °C and *p*_O_2__ = 0.2 bar. The energy to form an oxygen interstitial (int.) in the rocksalt layer is also shown.

The occupancies of all the oxygen sites were refined and it was observed that vacancies at room temperature were limited with a total oxygen stoichiometry of 9.96. These vacancies were confined to the O(1) site for the sample measured at 25 °C agreeing well with the DFT calculations (Fig. 6a). In cases where occupancies refined to 1.0, these were fixed in subsequent refinement cycles. As was expected the total oxygen stoichiometry decreased with increasing temperature, due to the reduction of the B-site cation, pointing towards the expected decrease in the B-site cation average oxidation state. The oxygen stoichiometry of 9.54 at 600 °C agrees well with previously published reports in related higher-order Ruddlesden–Popper materials by our group and others in the community.^16,18,19^ At 600 °C, the oxygen vacancies were observed in the O(1), O(2) and O(5) sites in the order O(1) ≈ O(5) < O(2) ≪ O(3) ≈ O(4), meaning that the vacancies predominantly prefer perovskite units of the structure.^48^ The atomic displacement parameters for the oxygen atoms were best fit with an anisotropic model and the thermal parameters show a considerable increase with the increase in temperature, which is expected (Table 3). The large *ab* plane anisotropy of the apical O(1) sites, particularly at high temperatures, points towards faster oxygen diffusion in these materials. This apical oxygen position is indeed known to exhibit an anisotropic Debye–Waller factor and has been widely reported for lower-order Ruddlesden–Popper K_2_NiF_4_ materials.^12,14,54^

To aid the neutron diffraction results, DFT calculations were used to investigate the formation energy of oxygen vacancies and oxygen interstitials. The energy to form an oxygen vacancy at each of the 5 distinct O sites (O1–O5) in the lowest energy La_2_Pr_2_Ni_3_O_10_ and end member La_4_Ni_3_O_10_ and Pr_4_Ni_3_O_10_ structures was calculated as shown in Fig. 6.

At 0 K, the oxygen interstitial is the lowest energy defect for all structures with a small negative formation energy for La_4_Ni_3_O_10_ (−0.127 eV) and Pr_4_Ni_3_O_10_ (−0.041 eV) and a small positive formation energy for La_2_Pr_2_Ni_3_O_10_ (0.067 eV). The formation energy for oxygen vacancies is above 1.5 eV for all sites indicating that there is a strong preference for interstitials in the (M)_4_Ni_3_O_10_ structure at low temperatures.

The sensitivity of the defect formation energies to the values of the *U* correction on Ni was tested by calculating the oxygen vacancy and interstitial formation energies without a *U* correction as shown in Fig. S4.† Consistent with Fig. 6a, the interstitial sites is the most favourable defect at 0 K. Without a *U* correction, the formation energy of all oxygen vacancies increased, and the O(2) site has a smaller formation energy than the O(1) site. However, the overall trend is consistent with the O(4) site in the rocksalt layer having the largest formation energy.

The variation in the formation energy of oxygen interstitials and O(1) oxygen vacancies as a function of temperature is shown in Fig. S5.† For the La_4_Ni_3_O_10_, La_2_Pr_2_Ni_3_O_10_ and Pr_4_Ni_3_O_10_ structures, the temperature below which oxygen interstitials have lower formation energy than O(1) vacancies are predicted to be 723, 708 and 795 K, respectively at *p*_O_2__ = 0.2 bar. This indicates that the defect chemistry of the La_*x*_Pr_4−*x*_Ni_3_O_10_ structures can be subtly tuned *via* the *x* composition and that the thermal history of the sample and cooling conditions, such as the oxygen partial pressure and cooling rate may have a large impact.

At the measurement temperature of 800 °C, oxygen vacancies are the lowest energy defects. The oxygen vacancy formation energy in Fig. 6b increases layer by layer from the centre of the perovskite layer O(1) to the apical oxygen within the rocksalt layer (O4) in the sequence: O(1) < O(2) < O(3) ≈ O(5) ≪ O(4). Importantly, from Fig. 6a and b, it can be seen that the oxygen site (O1–O5) has a significantly larger influence on the vacancy formation energy than the *x* composition in the La_*x*_Pr_4−*x*_Ni_3_O_10_ structure.

At 800 °C, the low oxygen vacancy formation energy of the O(1) sites (0.304–0.407 eV) for all structures indicates that these sites should have a significant concentration of oxygen vacancies, with a smaller fraction of vacancies on the O(2), O(3) and O(5) sites. This is consistent with the experimental results of the L2P2N3 composition discussed above. The neutron diffraction refinement further shows full occupancy for O(4) sites, again agreeing well with DFT calculations which show the highest oxygen vacancy formation energy for the O(4) site among the five oxygen sites. A difference in L2P2N3 composition is that while DFT calculations show a majority of vacancy concentration at the O(1) site, the neutron data refinement shows that O(1) and O(5) sites almost have the same oxygen vacancy concentration.

Taking into consideration the site occupancies, distances between these vacant O sites and the atomic displacement parameters, it is possible to envision the possible transport pathways in these materials. The distances between the oxygen atoms at 600 °C for L2P2N3 were found to be: 2.72 Å between O(5) sites; 2.72 Å between O(1) and O(2) sites and 2.77 Å between O(1) sites. This suggests predominant participation of fractionally vacant sites *via* pathways such as O(1)–O(2)–O(1), O(1)–O(1)–O(1) and O(5)–O(5)–O(2) with a possible preference to the transport pathways involving O(1) and O(5) sites because of the relatively shorter distances and high vacancy concentration at these sites. This preference for a curved oxygen transport pathway such as O(1)–O(1)–O(1) around the NiO_6_ octahedra is expected and in agreement with the published literature for materials such as La_0.3_Sr_2.7_CoFeO_7−*δ*_, LaSr_3_Co_1.5_Fe_1.5_O_10−*δ*_, LaSrCo_0.5_Fe_0.5_O_4−*δ*_ and La_1−*x*_Sr_*x*_BO_3_ (B = Cr, Mn, Fe and Co).^16,48,55,56^

Similarly, for the L3P1N3 and L1P3N3 compositions (Tables S3 and S4† respectively), neutron data shows oxygen vacancies are spread over O(1), O(2) and O(3) sites and oxygen sites O(4) and O(5) were fully occupied. This agrees with our DFT calculations which show the highest oxygen vacancy formation energy for O(4) and O(5) sites. However, in both compositions instead of the O(1) site being the most vacant site as per DFT calculations, neutron data shows O(3), another equatorial site, as the most vacant site.

Again, considering the distances between neighbouring O sites which at 800 °C for L3P1N3 were found to be: 2.74 Å between O(3) sites, 2.74 Å between O(1) sites and 2.72 Å between O(2) and O(1) sites. This suggests three major transport pathways; O(3)–O(3)–O(3), O(1)–O(1)–O(1) and O(1)–O(2)–O(1) with O(3)–O(3)–O(3) being the dominant pathway because of the considerable vacancies present at the O(3) oxygen site. Similarly, the distance between oxygen sites for L1P3N3 at 800 °C was found to be 2.73 Å between O(1) sites; 2.78 Å between O(1) and O(2) sites and 2.72 Å between O(2) and O(3) sites suggesting the predominant participation of O(1), O(2) and O(3) sites visa transport pathways such O(3)–O(2)–O(3) and O(1)–O(1)–O(1).

**Fig. 7:**
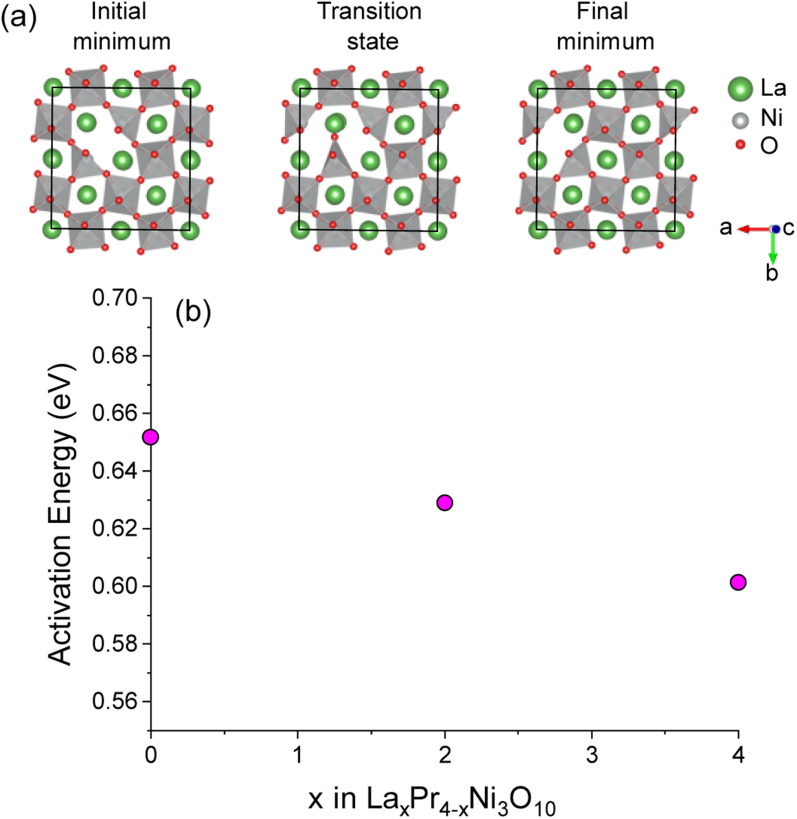
(a) Diagram of the initial and final minima and transition state along the O(1)–O(1) pathway in the perovskite layer of the La_32_Ni_24_O_79_ supercell structure. Only the central perovskite layer of the structure is shown for clarity. (b) Activation energies for O(1)–O(1) diffusion as a function of *x* composition in La_*x*_Pr_4−*x*_Ni_3_O_10_ calculated with NEB.

The activation energy for O(1)–O(1) vacancy diffusion was calculated using the CI-NEB approach for supercells of La_4_Ni_3_O_10_, Pr_4_Ni_3_O_10_ and the lowest energy convex hull structure of La_2_Pr_2_Ni_3_O_10_. The diffusion pathway of a vacancy between two O1 sites in the La_4_Ni_3_O_10_ structure is shown in Fig. 7a. The activation energy as a function of *x* in La_*x*_Pr_4−*x*_Ni_3_O_10_ is shown in Fig. 7b. The activation energy is lowest for the La_4_Ni_3_O_10_ (*x* = 0) end member (0.60 eV) and increases almost linearly as a function of *x* from La_2_Pr_2_Ni_3_O_10_ (0.63 eV) to Pr_4_Ni_3_O_10_ (0.65 eV). The very low activation barrier for O(1) oxygen vacancies within the centre of the perovskite blocks of the M_4_Ni_3_O_10_ structure is consistent with the low activation energies for O vacancy diffusion predicted computationally for the LaNiO_3_ structure (0.69–0.78 eV) in previous studies.^57,58^

## 4 Conclusions

Neutron diffraction studies and DFT calculations of three novel higher-order Ruddlesden–Popper phases were discussed. Across these newly synthesised compositions, it was observed that the lattice parameters show a systematic decrease with an increase in the Pr content of the composition, which agrees with the conception of effective ionic radii of Pr^3+^ being less than the corresponding La^3+^. L1P3N3 and L2P2N3 compositions were found to crystallise in the orthorhombic *Bmab*/*Fmmm* space group, with only L3P1N3 showing transition to higher symmetry tetragonal *I*4/*mmm* space group at 800 °C. L2P2N3 composition, however, was found to crystallise in monoclinic symmetry. It was also observed that orthorhombic strain in these materials increases with the increase in the Pr content of the composition. This is expected and is because of the decrease in ionic radii of Pr.

Lattice parameters were shown to increase linearly with the increase in temperature, with *c* parameter showing a significant increase than *a* and *b* cell parameters. This is attributed to the cumulative effect of stacking of layers along the *c* crystallographic direction and *c* axis being less restricted by the expansion of the rock-salt layers. A further observation of the *b* parameter showing only a slight increase as compared to *a* parameter is interpreted in terms of an increase in orthorhombic strain with the increase in temperature. The calculated TEC based on neutron diffraction measurements was found to be around 13.0 × 10^−6^ °C^−1^, which is of the same order as that of commonly used electrolyte materials in SOFC devices.

Furthermore, neutron diffraction and DFT calculations both suggested that vacancies prefer the perovskite block – O(1) and O(3) sites – and it, in fact, has been reported earlier that majority of the oxygen vacancies in these materials are confined to the perovskite layers, with a particular preference to equatorial oxygen sites. A further strong preference for the curved oxygen transport pathways such as O(1)–O(1)–O(1) around the NiO_6_ octahedra was recorded, in consonance with the earlier reports in these materials.

## Conflicts of interest

The authors declare that they have no known competing financial interests or personal relationships that could have appeared to influence the work reported in this paper.

## Supplementary Material

RA-013-D3RA01772A-s001
