# Supplementary material for: Neutron diffraction and DFT studies of oxygen defect and transport in higher-order Ruddlesden–Popper phase materials
Source: RSC Adv. 2023 May 5;13(20):13786–97. doi: 10.1039/d3ra01772a (PMC10160924; doi:10.1039/d3ra01772a)
Supplement: RA-013-D3RA01772A-s001 [file RA-013-D3RA01772A-s001.pdf]

Electronic Supplementary Material (ESI) for RSC Advances.
This journal is © The Royal Society of Chemistry 2023

Supplementary Information

Neutron Diffraction and DFT Studies of Oxygen Defect and Transport in Higher-Order Ruddlesden-Popper Phase Materials

Mudasir A. Yatoo[1,2], Ieuan Seymour[1] and Stephen J. Skinner[1,2]

[1]Imperial College London, Department of Materials, Faculty of Engineering, Exhibition Road, SW7 2AZ. United Kingdom
[2]EPSRC Centre for Doctoral Training in Advanced Characterisation of Materials, Exhibition Road, SW7 2AZ. United Kingdom

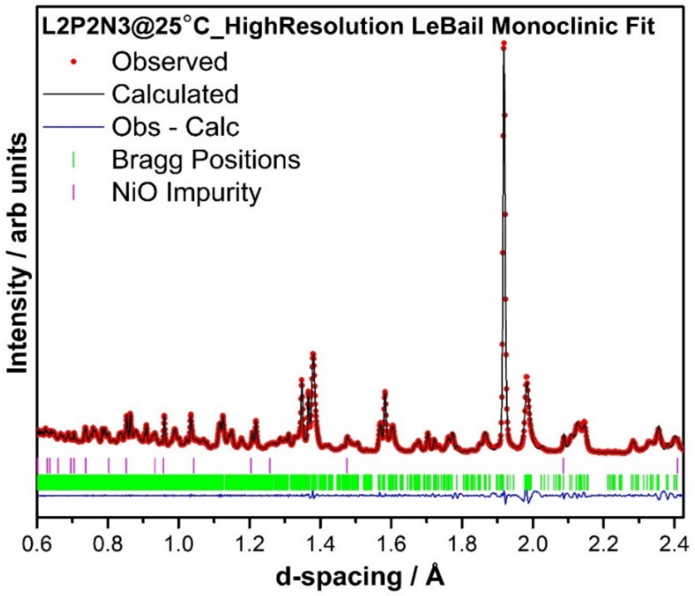

Figure S1. Le Bail refinement of the room-temperature high-resolution (bank 5) L2P2N3 data set in monoclinic $P12_1/a1$ space group (χ^2= 4.10; Rp = 1.7% and wRp = 1.1%).

Table S1. Fractional atomic coordinates and thermal parameters obtained after Rietveld refinement of L3P1N3 ND patterns recorded at 250˚C using orthorhombic model (Bmab).

**A**	**x**	**y**	**z**	***Uiso***
La/Pr(1)	0.0	-0.0043(2)	0.4328(2)	0.008(3)
La/Pr(2)	0.0	-0.0122(2)	0.2990(2)	0.013(6)
Ni(1)	0.0	0.0	0.0	0.008 (1)
Ni(2)	0.0	-0.0015(1)	0.1380(1)	0.009(8)
O(1)	0.25	0.25	0.0067(7)	
O(2)	0.0	0.9549(1)	0.0687(3)	
O(3)	0.25	0.25	0.135(3)	
O(4)	0.0	0.0377(2)	0.2134(3)	
O(5)	0.25	0.75	0.1457(3)	

Table S2. Fractional atomic coordinates and thermal parameters obtained after Rietveld refinement of L3P1N3 ND patterns recorded at 800˚C using tetragonal model (I4/mmm).

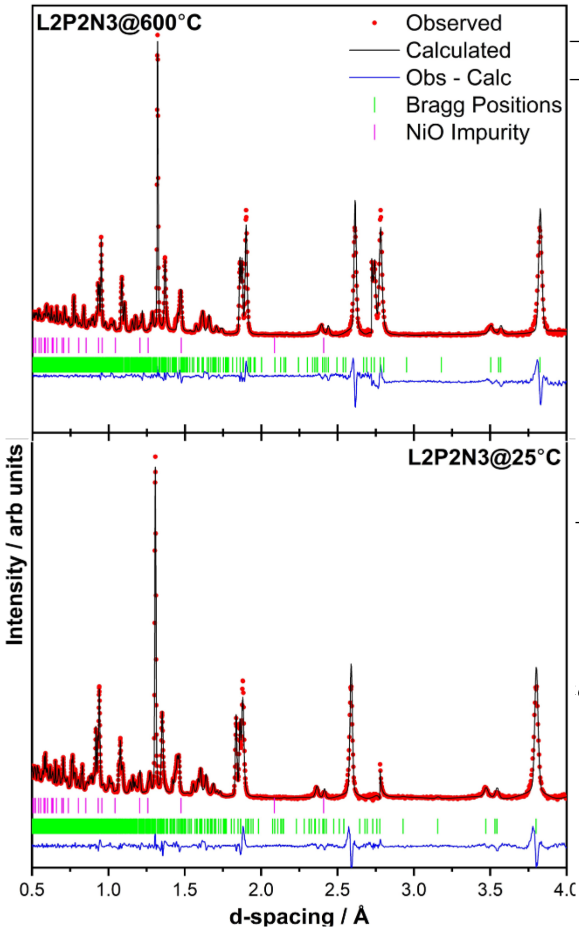

Figure S2. Rietveld refinement of the two data sets obtained at 25˚C (χ^2= 1.91; Rp = 6.5% and wRp = 3.0%) and 600˚C (χ^2= 1.52; Rp = 5.7% and wRp = 2.7%). A ~2% impurity of NiO was detected and was refined in cubic Fm3m space symmetry.

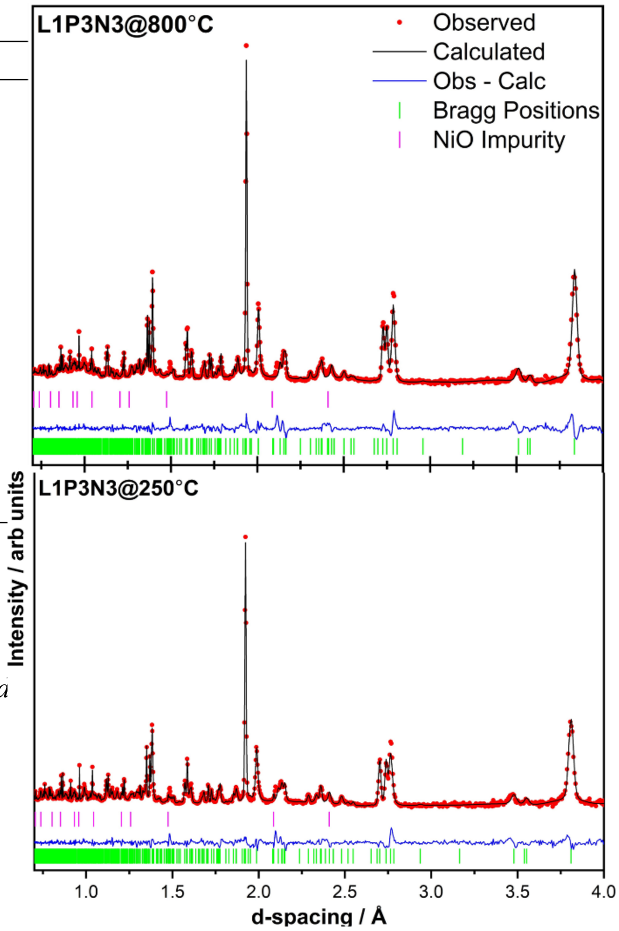

Figure S3. Rietveld refinement of the two data sets obtained at 250˚C (χ^2= 11.47; Rp = 8.7% and wRp = 7.6%) and 800˚C (χ^2= 14.36; Rp = 6.9% and wRp = 5.9%). A ~2% impurity of NiO was detected and was refined in cubic Fm3m space symmetry.

Atom	Atomic displacement parameters (Å^2) *x* 100			Fractional occupancy
	U_{11}/U_{iso}	***U_{22}***	***U_{33}***	
La(1)	1.9(3)			0.77(3)
Pr(1)	1.9(3)			0.33(3)
La(2)	1.8(7)			0.86(5)
Pr(2)	1.8(7)			0.14(5)
O(1)	0.7(6)	6.6(3)	11.0(4)	0.97(3)
O(2)	5.4(8)	5.4(8)	3.8(9)	0.97(3)
O(3)	0.6(9)	3.5(1)	3.5(1)	0.93(2)
O(4)	6.3(3)	6.3(3)	1.6(1)	1.0

Table S4. Rietveld refinement parameters obtained for the L1P3N3 composition recorded at 800˚C

Atom	Atomic displacement parameters (Å^2) *x* 100					Fractional occupancy
	U_{11}/U_{iso}	***U_{22}***	***U_{33}***	***U_{12}***	***U_{23}***	
La(1)	3.5(3)					0.32(6)
Pr(1)	3.5(3)					0.68(6)
La(2)	1.7(4)					0.39(7)
Pr(2)	1.7(4)					0.61(7)
O(1)	6.8(1)	5.6(1)	1.7(1)	6.1(8)		0.97(5)
O(2)	5.3(8)	0.8(7)	3.9(8)		1.0(8)	0.96(3)
O(3)	0.5(6)	3.1(1)	2.6(1)	0.6(6)		0.83(5)
O(4)	3.5(1)	11.2(1)	3.5(7)		3.3(1)	1.0
O(5)	1.3(7)	1.9(5)	2.1(7)	0.3(4)		1.0

with orthorhombic model (Bmab).

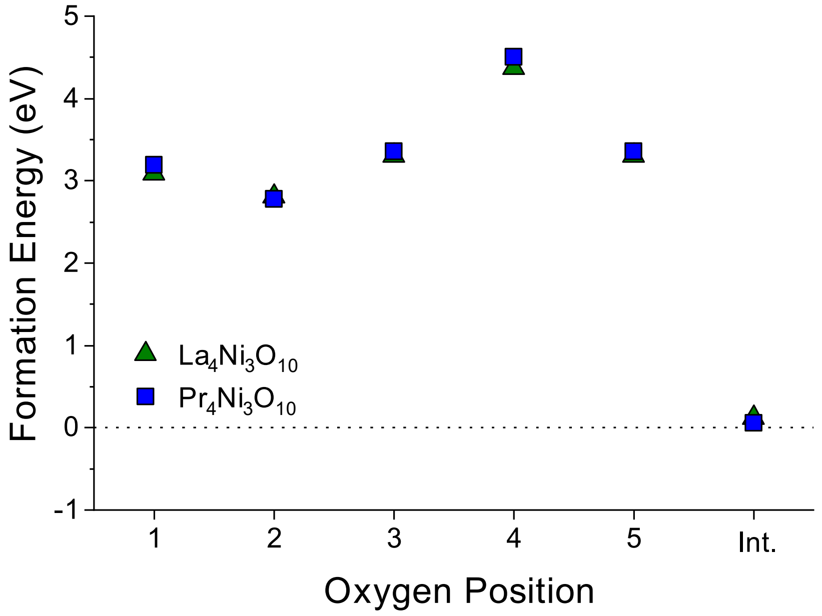

Figure S4: Plot of formation energy for oxygen vacancies at the O1–O5 sites in the Bmab primitive cell structures of $La_4Ni_3O_{10}$ and $Pr_4Ni_3O_{10}$, with 2 formula units per cell ($M_8Ni_6O_{20\pm1}$) at T = 0 K without a U correction on Ni.

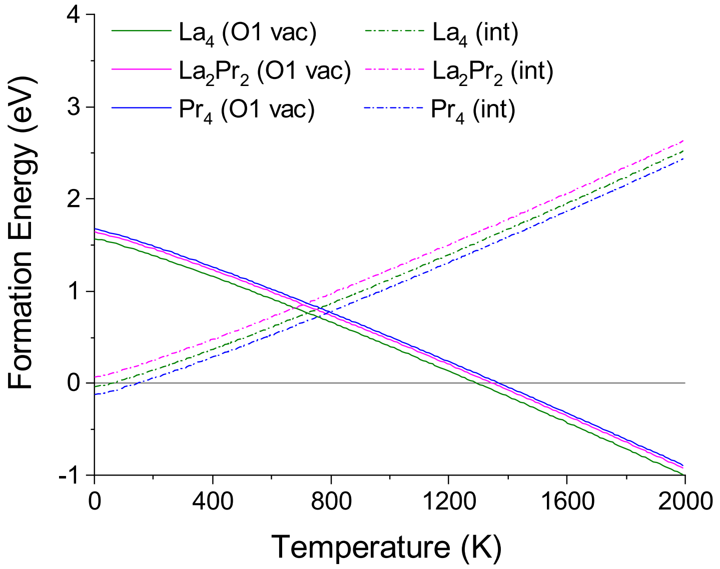

Figure S5. Variation in oxygen interstitial (int.) and O1 vacancy formation energy as a function of temperature for lowest energy $La_4Ni_3O_{10}$, $La_2Pr_2Ni_3O_{10}$ and $Pr_4Ni_3O_{10}$ structures.
